# Construction Notes

**Published:** 1897-01-23

**Authors:** 


### CONSTRUCTION NOTES.

### New Fever Hospital at Edinburgh.

The following plans for the new Fever Hospital at Edinburgh have been approved and accepted by the convener of the Public Health Committee. Within the principal entrance on the left is the porter's lodge, and a little further in, on the right, the medical superintendent's house. Further within, and facing the principal entrance, are the general offices, and in the rear of these the stores, kitchen and dining-room block; while beyond are the nurses' and servants' homes. These buildings occupy a position nearly central in the hospital grounds, the ward pavilions being arranged in double rows to east and west—those on the east being entirely for scarlet fever, and those on the west for the other diseases to be provided for—viz., diphtheria, typhoid, and erysipelas in the north-western; and measles, chicken-pox, whooping cough, and typhus in the south-western ranges. Reception and observation wards—the latter for undecided cases—are placed on each side near the principal entrance, and isolation wards for complicated cases towards the further extremities of the respective groups. At the north-east corner of the grounds is the ambulance station. Near this are lecture-rooms, pathological laboratory, museum, and mortuary buildings; further south are laundry, boiler, disinfector, electrical power, and incinerator buildings. Ample open space is left for separate recreation grounds in convenient proximity to the pavilions for the several classes of disease, besides airing courts between the pavilions. The wards are arranged in separate pavilions, one or more for each class of disease, according to the number of patients to be provided for, and mostly of two storeys—the stair in every case being so placed as entirely to cut off all direct communication between the lower and upper wards and offices. The pavilions are placed so as to get as much sun as possible—that is, with their length north and south. The floors are intended to be of fire-resisting construction. The heating will be by means of ventilating steam coils, supplemented by central ventilating stoves in the general wards, the kitchen, and dining-room block, which occupies a central position on the estate. To the south of this block will be the servants' and nurses' homes, the former two storeys in height, with rooms for 100 nurses. For access to the various parts of the premises, ambulance drives are provided from the principal entrance. Adjoining the ambulance station the plan shows a building for educational purposes, and near it is a pathological laboratory and museum building.

